# Microenvironmental stiffness mediates cytoskeleton re-organization in chondrocytes through laminin-FAK mechanotransduction

**DOI:** 10.1038/s41368-022-00165-5

**Published:** 2022-03-11

**Authors:** Chenchen Zhou, Mengmeng Duan, Daimo Guo, Xinmei Du, Demao Zhang, Jing Xie

**Affiliations:** 1 State Key Laboratory of Oral Diseases, West China Hospital of Stomatology, Sichuan University, Chengdu, China; 2 National Clinical Research Center for Oral Diseases, West China Hospital of Stomatology, Sichuan University, Chengdu, China; 3 Department of Cariology and Endodontics, West China Hospital of Stomatology, Sichuan University, Chengdu, Sichuan China

**Keywords:** Biological fluorescence, Immunoblotting

## Abstract

Microenvironmental biophysical factors play a fundamental role in controlling cell behaviors including cell morphology, proliferation, adhesion and differentiation, and even determining the cell fate. Cells are able to actively sense the surrounding mechanical microenvironment and change their cellular morphology to adapt to it. Although cell morphological changes have been considered to be the first and most important step in the interaction between cells and their mechanical microenvironment, their regulatory network is not completely clear. In the current study, we generated silicon-based elastomer polydimethylsiloxane (PDMS) substrates with stiff (15:1, PDMS elastomer vs. curing agent) and soft (45:1) stiffnesses, which showed the Young’s moduli of ~450 kPa and 46 kPa, respectively, and elucidated a new path in cytoskeleton re-organization in chondrocytes in response to changed substrate stiffnesses by characterizing the axis shift from the secreted extracellular protein laminin β1, focal adhesion complex protein FAK to microfilament bundling. We first showed the cellular cytoskeleton changes in chondrocytes by characterizing the cell spreading area and cellular synapses. We then found the changes of secreted extracellular linkage protein, laminin β1, and focal adhesion complex protein, FAK, in chondrocytes in response to different substrate stiffnesses. These two proteins were shown to be directly interacted by Co-IP and colocalization. We next showed that impact of FAK on the cytoskeleton organization by showing the changes of microfilament bundles and found the potential intermediate regulators. Taking together, this modulation axis of laminin β1-FAK-microfilament could enlarge our understanding about the interdependence among mechanosensing, mechanotransduction, and cytoskeleton re-organization.

## Introduction

It is well recognized that microenvironmental mechanics mediate cell behaviors, control cell differentiation, and determine cell fate^1,2^. The stiffness of extracellular matrix (ECM) is one of the key microenvironmental biomechanical factors, which plays an indispensable role in organ development^3^, tissue maturation^4^ and disease occurrence^5^. Recent research progress has begun to reveal the important regulatory role of microenvironmental stiffness on cell behaviors based on different kinds of cell types. For stem cells, microenvironmental stiffness could direct the multiple differentiations toward osteogenesis, chondrogenesis, adipogenesis and neurogenesis of bone marrow stem cells (BMSCs)^2,6^, adipose-derived stromal cells (ASCs)^7^, and odontogenic stem cells^8,9^. For adult cells, microenvironmental stiffness could mediate cell proliferation^10^, migration^11^, adhesion^12^, cell cycling^13^, and cell-to-cell communication^14^. The general biomechanical mechanism on how the stiffness regulates cell behaviors is that cells could actively sense microenvironmental stiffness and couple this stiffness to the actin cytoskeleton via multi-transmembrane complexes comprising integrins, receptor tyrosine kinases, and cadherins^15,16^. Cells firstly exert contraction forces onto the microenvironmental substrate and subsequently adjust their cell-ECM adhesion strength via the changes of focal adhesion plaques (FA), and finally reach a homeostasis between intracellular forces based on cytoskeletal contractility and extracellular forces coupling to the ECM stiffness^17,18^. Although many reports have begun to elucidate the mechanosensory bio-mechanisms based on secreted ECM proteins, ion channel proteins, membrane receptor proteins, and adhesion complex proteins, a complete picture of biomechanical mechanism has yet to be established.

Articular cartilage is the typical connective tissue affected by various external forces^19^. It acts as a shock absorber and load distributer at the weight bearing interfaces of the joints and functions to withstand and redistribute the tensile, compressive and shear stress imposed by the whole body^20,21^. This strong pressure resistant capacity depends on its unique cellular and special extracellular matrix components. Chondrocytes, as the unique cells in all types of cartilages, are regarded to be mechanosensitive cells and can respond to various mechanical stresses throughout life^22,23^. They are also responsible for the integrity maintenance of extracellular matrix, which is composed of structural components including proteoglycan and collagen II, and linkage proteins involving fibronectins and laminins^24,25^. Proteoglycan and collagen II interact with linkage proteins to establish an extracellular network that allows cartilage to resist various compressive loads.

ECM stiffness has been identified as a vital contributor in mediating chondrocyte proliferation^26^, differentiation^27^, and redifferentiation^28^. In the previous reports, we have confirmed the characteristics of chondrocyte mechanoresponses^29^, cell morphology control^30^, cell contractile function^31^, and cell-to-cell communication^14^. In the current study, by using substrates with different stiffnesses fabricated by silicon-based elastomer polydimethylsiloxane (PDMS), we propose a novel mediating axis in cytoskeleton re-organization of chondrocytes from secreted extracellular protein, focal adhesion plaque to microfilament bundling. With the help of molecular biology technologies including scanning electron microscope, RNA sequencing, and immunomolecular experiments, we aim to enlarge the understanding about the interdependence among mechanosensing, mechanotransduction, and cytoskeleton re-organization.

## Results

### Cell morphology changes in chondrcoytes in response to stiff and soft substrates

**Fig. 1:**
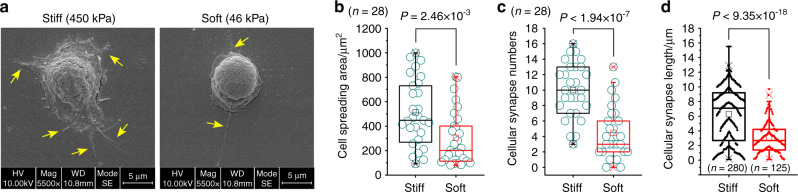
Cell morphology changes of chondrocytes in response to PDMS substrates with different stiffnesses. **a** Representative SEM images showing the cell morphology changes of chondrocytes seeded onto PDMS substrates with stiff (450 kPa) and soft (46 kPa) stiffnesses. Yellow arrows indicates cellular synapses. **b** Box and whisker plot showing the changes of cell spreading areas of chondrocytes seeded onto PDMS substrates with stiff (450 kPa) and soft (46 kPa) stiffnesses. Twenty-eight cells per group from eight independent experiments were used to calculate the cell spreading areas. **c** Box and whisker plot showing the changes of cellular synapses of chondrocytes seeded onto PDMS substrates with stiff (450 kPa) and soft (46 kPa) stiffnesses. Twenty-eight cells per group from eight independent experiments were used to calculate the cellular synapses. **d** Box and whisker plot showing the changes of cellular synapse lengths of chondrocytes seeded onto PDMS substrates with stiff (450 kPa) and soft (46 kPa) stiffnesses. Two hundred and eighty cellular synapses of twenty-eight cells from the stiff group and one hundred and twenty-five cellular synapses of twenty-eight cells from the stiff group were applied to calculate the cellular synapse lengths. The SEM experiments are based on eight independent experiments (*n* = 8). The data in (**b**), (**c**), & (**d**) are shown in the box (from 25%, 50% to 75%) and whisker (minimum to maximum values) plots. All significant data presented in (**b**), (**c**) & (**d**) are based on two-tailed Student’s *t*-tests.

To explore the cell morphology changes of chondrocytes in response to substrates with different stiffnesses, we firstly fabricated two kinds of PDMS substrates with different Young’s moduli (stiff, 450 kPa, 15: 1, and soft, 45 kPa, 45:1 (PDMS elastomer v.s. curing agent)) as previously reported^8,32^. We seeded chondrocytes onto the PDMS substrates to explore the cell behavior changes of chondrocytes. After cell attachment for 24 h, we observed the difference in cell morphology by SEM (Fig. 1a). We found that chondrocytes showed a larger spreading area in the stiff group than in the soft group. Moreover, chondrocytes showed more cellular synapses in the stiff group compared to those in the soft group. Based on eight independent experiments, we randomly chose twenty-eight cells from SEM experiments to analyze the changes of spreading areas and cellular synapses. We found that cell spreading areas of chondrcoytes in the stiff group were generally larger than those in the soft group by box and whisker plot (Fig. 1b). The mean cell spreading area in the stiff group was approximately 450 μm^2^ and it meant that the average cell diameter reached to be approximately 20 μm (Supplementary material-Data source-Fig.1b), while in the soft group, The mean cell spreading area in the stiff group was approximately 200 μm^2^ which meant the average cell diameter was below 16 μm (Supplementary material-Data source-Fig.1b). We next analyzed the changes of cellular synapses. The results showed that cellular synapse number per cell was significantly more in the stiff group than that in the soft group (Fig. 1c), and furthermore, the mean length of cellular synapses in the stiff group were longer compared to those in the soft group (Fig. 1d).

### The difference of laminin β1 enrichment in chondrcoytes in response to stiff and soft substrates

**Fig. 2:**
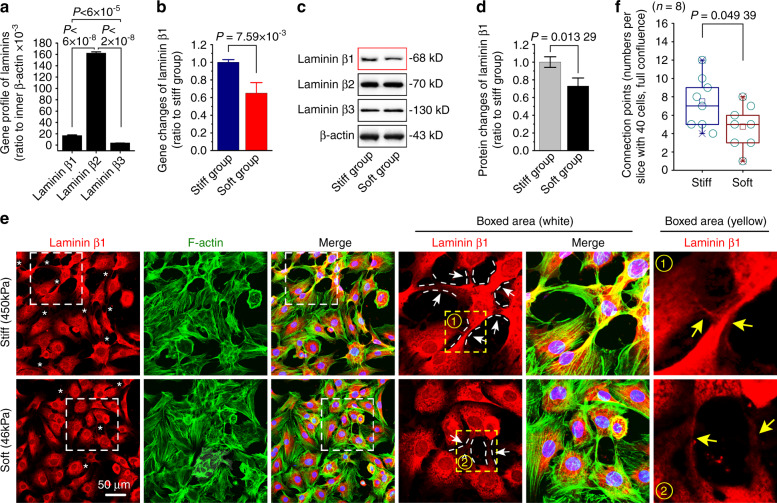
Expressions of laminin β1 in chondrocytes in response to PDMS substrates with different stiffnesses. **a** RNA sequencing indicating the basal gene expression levels of laminin beta family in chondrocytes. Their expressions were all ratio to the inner β-actin gene. **b** qPCR showing mRNA changes of laminin β1 in chondrocytes in response to stiff and soft substrates. β-actin gene was used as the inner control. **c** Western blotting indicating the protein changes of laminin β1 in chondrocytes in response to stiff and soft substrates. **d** OD quantification confirming the protein changes of laminin β1 in chondrocytes in response to stiff and soft substrates. **e** Immunofluorescence by CLSM showing the expressions of laminin β1 in chondrocytes in response to stiff and soft substrates. *indicates the expressions of laminin β1 at the site of connections between the two cells. Arrows further indicate the details of laminin β1 at the site of connections between the two cells.The experiments in (**a**), (**b**), (**c**), & (**e**) are all based on three independent experiments (*n* = 3). The data in F are shown in the box (from 25%, 50% to 75%) and whisker (minimum to maximum values) plots. All significant data presented in (**a**), (**b**), (**d**), and (**f**) are based on two-tailed Student’s *t*-tests.

**Fig. 3:**
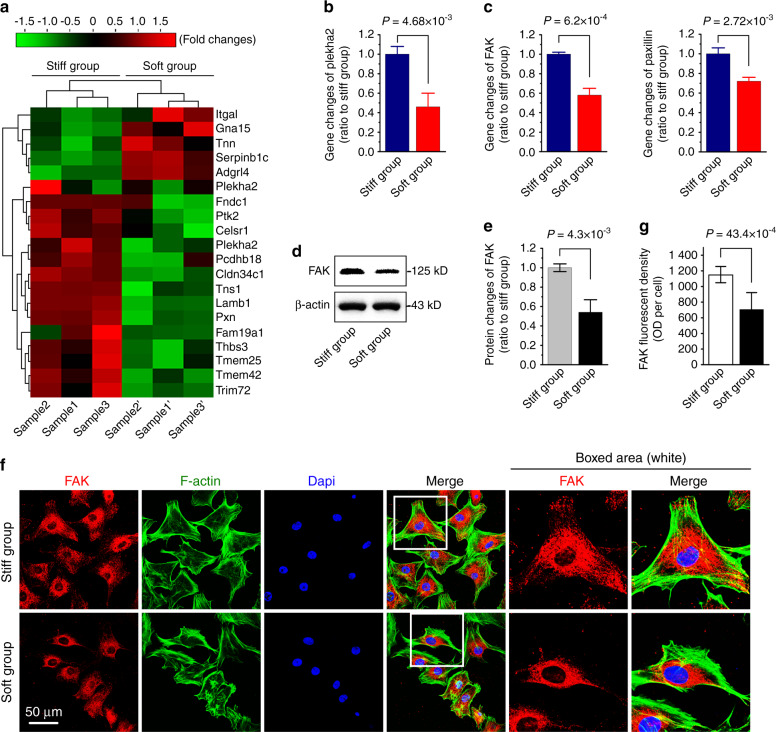
Expression profile of extracellular proteins/focal adhesion proteins in chondrocytes in response to PDMS substrates with different stiffnesses. **a** Pheatmap by RNA sequencing indicating the mRNA changes of extracellular proteins/focal adhesion proteins in chondrocytes in response to stiff and soft substrates. Cell layste samples were collected at 72 h after being seeded onto stiff and soft substrates. Data were presented as FPKM (Fragments Per Kilobase of transcript per Million fragments mapped) by online R-package from RNA sequencing data. Three independent pairs of samples, i.e., Sample 1 and 1’, Sample 2 and 2’, and Sample 3 and 3’, were from the same mother cells, respectively. **b**, **c** qPCR confirming the mRNA changes of plekha2, FAK and pxn in chondrocytes in response to stiff and soft substrates. β-actin gene was used as the inner control. **d** Western blotting showing the FAK protein in chondrocytes in response to stiff and soft substrates. **e** OD quantification confirming the protein changes of FAK in chondrocytes in response to stiff and soft substrates. **f** Immunofluorescence by CLSM showing the expressions of FAK in chondrocytes in response to stiff and soft substrates. **g** Fluorescent OD quantification confirming the protein changes of FAK in chondrocytes in response to stiff and soft substrates. The experiments in (**b**), (**c**), & (**f**) are all based on three independent experiments (*n* = 3). All significant data presented in (**b**), (**c**), (**e**), & (**g**) are based on two-tailed Student’s *t*-tests.

Cartilage extracellular matrix mainly consists of two kinds of proteins. One kind is structural component proteins including collagen II and proteoglycan, and the other kind is linkage proteins including laminins and fibronectins, which are responsible for sensing and transmission of extracellular signals^24,25^. From our RNA sequencing, we observed that there were three types of laminin betas expressed in chondrocytes (Fig. 2a) and only laminin β1 was changed in chondrocytes in response to different substrate stiffnesses (Lamb1, Fig. 3a). We re-checked the mRNA expressions of laminin betas by qPCR and found the mRNA of laminin β1 was changed in response to different stiffnesses (Fig. 2b) but laminin β2 and β3 were not changed (Supplementary Fig. S1). We performed western blotting to detect the changes of laminin betas at protein level in chondrocytes in response to substrate stiffness and found that only protein laminin β1 was changed (Fig. 2c, d) and the protein expressions of laminin β2 and β3 showed no significant changes (Fig. 2c and Supplementary Fig. S2). We then examined the distributions of laminin β1 in chondrocytes with a high cell density (close to full confluence (> 90%) due to the secreted extracellular protein) by using immunofluorescence and CLSM. We found that a large amount of laminin β1 (including pro-protein) was highly expressed, moreover, laminin β1 could form more connections (linkages) between chondrocytes on the stiff group at the same cell density (Fig. 2e, *indicated and boxed area showed). The quantitative analysis of connections further confirmed this result (Fig. 2f).

### Changes of core focal adhesion protein FAK in chondrcoytes in response to stiff and soft substrates

As it is well recognized that extracellular mechanical signals could be transmitted into the cell by triggering the focal adhesion plaques^33,34^. Thus, we screened the mRNA changes of extracellular proteins/focal adhesion proteins in chondrocytes in response to stiff and soft substrates by RNA sequencing and formed a pheatmap by online R-package (Fig. 3a). We found that pleckstrin homology domain containing A2 (plekha2), which was responsible for the intracellular binding of fibronectins and laminins^35^, was changed in chondrocytes in response to substrate stiffnesses. We also found focal adhesion kinase (FAK, also known as ptk2) and paxillin (pxn), an adapter of focal adhesion which works as the partner of FAK^36^, were significantly changed. Thus, we next used qPCR and confirmed the changes of these genes (Fig. 3b, c). We then used western blotting to detect the change of FAK at the protein level (Fig. 3d, e). To further explore the distribution of FAK in chondrocytes, we performed immunofluorescence. By using CLSM we found that the expression and distribution of FAK on the stiff substrate were stronger than those on the soft substrate (Fig. 3f). The immunofluorescent OD quantification further confirmed this result (Fig. 3g).

### Interaction between secreted extracellular laminin β1 and focal adhesion protein FAK

**Fig. 4:**
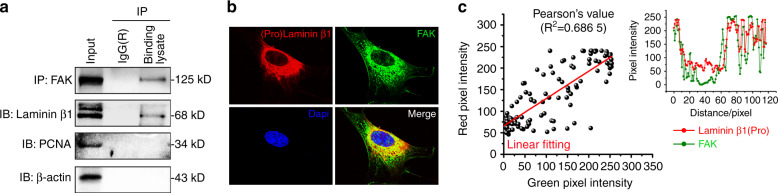
Interaction between the secreted extracellular protein laminin β1 and focal adhesion protein FAK. **a** Co-IP showing the binding between laminin β1 and FAK in chondrocytes. FAK was chosen to be the bait protein and laminin β1 was the prey protein. Nuclear protein PCNA (Proliferating cell nuclear antigen) was chosen to be a negative prey protein. β-actin was not only an internal control, but also a constituent unit of cytoskeleton. **b** Immunofluorescence by CLSM showing the co-expressions of laminin β1 (pro-protein) and FAK in chondrocytes. Laminin β1 was shown to be red fluorescent light and FAK was to be green fluorescent light. The objective lens was selected to be 100 × (Oil immersion lens). **c** Co-location analysis indicating the co-expressed relation between laminin β1 (pro-protein) and FAK in chondrocytes. The expression of co-distribution between laminin β1 (pro-protein) and FAK was achieved by linear fitting (Pearson’ *R* value). All experiments are based on three independent experiments (*n* = 3). The presentation of images in (**a**) & (**b**) are chosen as the representative images.

To further explore whether there was direct interaction between laminin β1 and FAK, we performed Co-IP (Fig. 4a). The result indicated that secreted extracellular linkage protein, laminin β1, had a direct binding with the focal adhesion protein, FAK. We then made further exploration by using colocalization analysis between pro-laminin β1 (intracellular pro-protein) and FAK. We found that, in cytoplasm, there was a co-distribution relationship between intracellular pro-protein of laminin β1 (pro-protein of extracellular active proteins) and FAK (Fig. 4b, c), although the linear fit of the two proteins was not much high (R^2^ = 0.686 5). Taking together, these results indicated chondrocytes might directly transmit mechanical signals to focal adhesion plaques through the interaction between laminin β1 and FAK, which could initiate the first intracellular step in signal transduction cascade^34^.

### Cytoskeleton re-organization in chondrcoytes in response to stiff and soft substrates

**Fig. 5:**
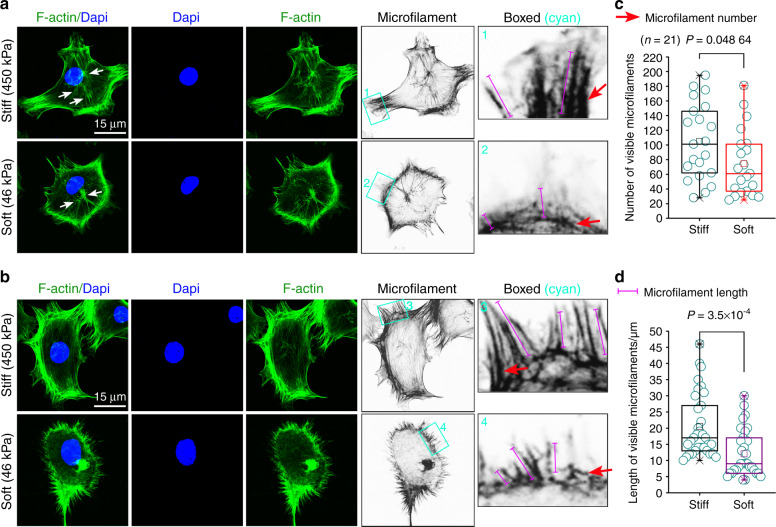
Detailed changes of cytoskeleton in chondrocytes in response to PDMS substrates with different stiffnesses. **a**, **b** Immunofluorescence by CLSM showing the expressions of F-actin in chondrocytes in response to stiff and soft substrates. White arrows indicated formation of microfilaments (F-actin bundle) near the nucleus. Grayscale images were used to indicate the changes of microfilaments in chondrocytes in response to stiff and soft substrates. Cyan boxed areas indicated the differences in microfilament number and length. **c** Quantitative analysis indicating the changes of microfilament numbers in chondrocytes in response to stiff and soft substrates. **d** Quantitative analysis indicating the changes of microfilament lengths in chondrocytes in response to stiff and soft substrates. The experiments of F-actin immunofluorescent staining by CLSM are based on at least 20 independent experiments (*n* = 20). The data in (**c**) & (**d**) are shown in the box (from 25%, 50% to 75%) and whisker (minimum to maximum values) plots. All significant data presented in (**c**) & (**d**) are based on two-tailed Student’s *t*-tests.

It was reported that FAK plays an important role in cytoskeleton formation^35^. We further explored the cytoskeleton changes in chondrocytes in response to substrate stiffness. From a number of independent repeated experiments (*n* > 20), we observed that there were two types of cytoskeletons, with the most obvious differences, in chondrocytes in response to substrate stiffness. One type was that can form microfilament bundles (F-actin) near the nuclei of chondrocytes (Fig. 5a, white arrows indicated). These cells showed longer and broader microfilament bundles and larger cell spreading area in the stiff group than those in the soft group. The other type was that can form microfilaments only at the boundary of cell membrane of chondrocytes (Fig. 5b). The proportion of these cells in the second type was higher than the first one. From the typical images presented in Fig. 5b, we could observe that cells can form a wide circle of microfilament bundles at the boundary of their cell membranes on the stiff substrate, while on the soft substrate, the microfilament bundles showed an outward emission state, but they were very short and thin. We further analyzed and quantified the number and length of microfilament bundles (Fig. 5c, d). The results confirmed that more and longer microfilament bundles of chondrocytes were shown on the stiff substrate than those on the soft substrate.

**Fig. 6:**
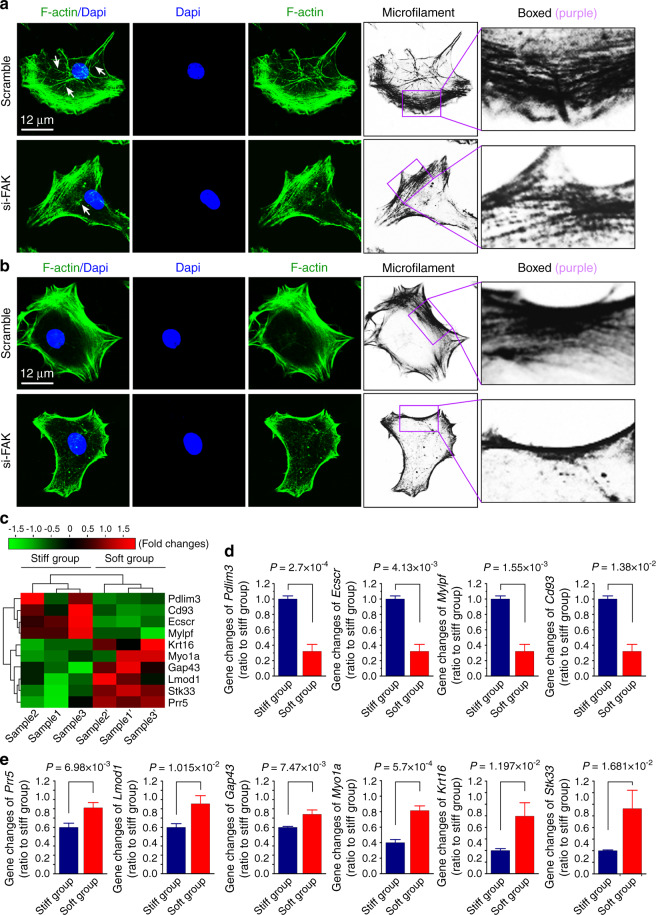
The potential protein mediators participated in re-organization of cytoskeleton in chondrocytes in response to PDMS substrates with different stiffnesses. **a**, **b** Immunofluorescence by CLSM showing the formation and distribution of microfilaments (F-actin bundle) in chondrocytes after reduction of FAK by siRNA interference. White arrows indicated formation of microfilaments near the nucleus. Grayscale images were used to indicate the changes of microfilaments in chondrocytes in response to stiff and soft substrates. Purple boxed areas indicated the differences in microfilament bundle. **c** Pheatmap by RNA sequencing indicating the mRNA changes of protein mediators in chondrocytes in response to stiff and soft substrates. Cell layste samples were collected at 72 h after being seeded onto stiff and soft substrates. Data were presented as FPKM (Fragments Per Kilobase of transcript per Million fragments mapped) by online R-package from RNA sequencing data. Three independent pairs of samples, i.e., Sample 1 and 1’, Sample 2 and 2’, and Sample 3 and 3’, were from the same mother cells, respectively. **d** qPCR confirming the mRNA down-regulation of protein mediators in chondrocytes in response to soft substrates relative to the stiff group. **e** qPCR confirming the mRNA up-regulation of protein mediators in chondrocytes in response to soft substrates relative to the stiff group. All experiments by qPCR in (**d**) & (**e**) are based on three independent experiments (*n* = 3). All significant data presented in (**d**) & (**e**) are based on two-tailed Student’s *t*-tests.

From Co-IP in Fig. 4a, we could also found that FAK and β-actin had no direct binding. It meant that FAK, as a kinase, may modulate cytoskeleton re-organization through a series of intermediate regulatory proteins^34^. We firstly used siRNA to knockdown the expression of FAK in chondrocytes and found that reduction of FAK could disturb the organization of cytoskeleton in both the first type cells with the formation of microfilaments (F-actin bundle) near the nuclei (Fig. 6a) and the second type cells with the formation of microfilaments only at the boundary of cell membrane (Fig. 6b). Furthermore, we dug out the potential protein mediators, which could be triggered by FAK and modulate cytoskeleton re-organization in chondrocytes, from the database of RNA sequencing. We found that there were ten protein mediator candidates participated in this process (detailed information could be seen in Supplementary material-Data source-Fig. 6a), and we presented these candidates with a pheatmap by online R-package (Fig. 6c). Among them, four protein mediators were highly expressed and the other six protein mediators were lowly expressed in the stiff group relative to those in the soft group. We finally performed qPCR and confirmed these highly expressed mediators (Fig. 6d) and lowly expressed ones (Fig. 6e) in the stiff group relative to those in the soft group.

## Discussion

**Fig. 7:**
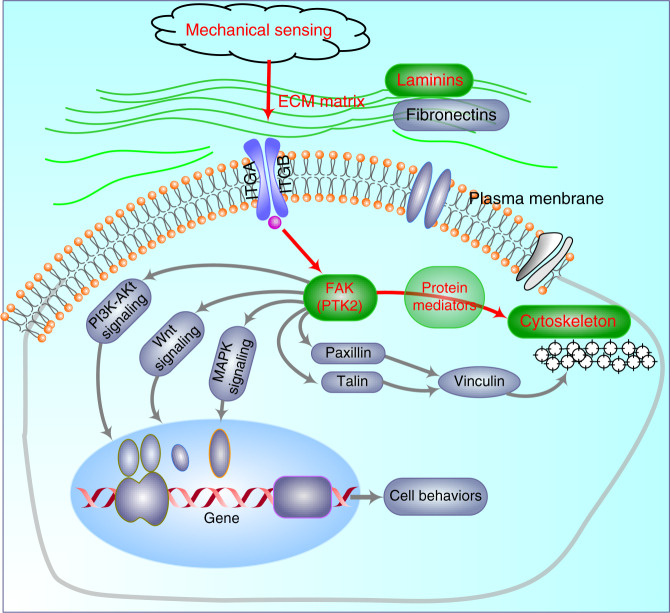
The schematic diagram showing the mediation axis from mechanical sensing, extracellular laminin β1 forming, focal adhesion protein FAK changing to cytoskeleton re-organization. Mechanical signals enter the chondrocytes to change the focal adhesion proteins, and then initiate the activation of signaling network, and finally regulate a variety of biological behaviors of the chondrocytes. The gray networks in the biomechanical regulation are involved but not presented in the current study; The red axis indicates the mediation process in the current study.

A lot of evidence has indicated the importance of the physical stiffness of ECM on cell fate controls, especially for those cells that are sensitive to mechanics or live in a mechanical environment for a lifetime^1–3,37^. To study the response of chondrocytes to microenvironmental mechanics is not only because chondrocytes are mechanical sensitive cells, but also because of the challenges in the repair of cartilage after mechanical injury^38^. Chondrocytes can change their intrinsic protein expressions of secreted extracellular proteins, phenotypic regulatory proteins, and signal pathway proteins to adapt the changes of external mechanical stiffness^39^. In this process, chondrocytes firstly exert contraction forces onto the substrates and correspondingly adjust their cell-to-ECM adhesion strength by the changes of composition and size of focal adhesion complex proteins^7,35^. It is well known that focal adhesion complex proteins (focal adhesion plaque, FA) play a core role in sensing the external forces^34^ and their changes subsequently trigger cell responses which are directly linked to the cellular biophysical changes including cytoskeleton re-organization and cellular chemical changes involving the initiation of cytoplasmic signaling cascades^35^. In our current study, we elucidated one of the mediation processes in cytoskeleton re-organization by the axis of laminin-FAK-microfilament (Fig. 7). Although this was just only one of many regulatory paths in cytoskeleton re-organization, we indicated its importance and enlarged the understanding in cellular biophysical changes triggered by microenvironmental stiffness.

Laminins, an important family of extracellular matrix glycoproteins, are considered to be the major non-collagenous component of basement membranes/the extracellular linkage proteins^40^. They have functionally participated in many cellular biological processes involving cell adhesion, stem cell differentiation, migration, and cellular synapse formation^41^. Three non-identical chains, namely laminin alpha, beta, and gamma, constitute the whole family^42^. The family members expressed in the chondrocytes by RNA sequencing include laminin alpha 1~5, laminin beta 1~3, and laminin gamma 1~2 in the current study. The highest expressed member is laminin β2, but the one with statistical difference in chondrocytes in response to substrate stiffness is laminin β1. From the immunofluorescent staining of laminin β1, we could find that it is easier to form connections between chondrcoytes on the stiff substrate than those in the soft substrate. However, the expression pattern of laminin β1 was different from the fibronectin1, a member of another linkage protein family fibronectins^43^. Specifically, laminin β1 presented in chondrocytes in the form of a large number of pro-proteins, but fibronectin 1 was mainly expressed outside chondrcoytes, especially in the gap between the two cells^14^. This different expression patterns between laminins and fibronectins in chondrocytes might determine the different functions although both of them belonged to the linkage proteins.

Two main adhesion complexes participate in the mechanosensitive interactions between cells and matrix. one is focal adhesion complexs (FAs) and the other is adherens junctions (AJs). AJs are mainly involved in cell-to-cell interactions^36^, while FAs function to sense external forces exerted by ECM^33^. FAs sense the external mechanical signaling and react to these signaling by simultaneously achieving its direct feedback to the cytoskeletal complexes and triggering the initiation of the cytoplasmic signaling transmission into downstream targets^34^. FAs can be anchored to the extracellular matrix (pericellular matrix) by their receptors and transmembrane proteins, such as integrin family, and are attached to the cytoskeleton by direct or indirect protein linkages^35^. Thus, they serve as the core bridge between ECM component and cytoskeleton. FAK, as a core kinase in FAs, it could directly interact with laminin β1 in chondrocytes as shown in the current study, although there might be intermediate carrier proteins including membrane receptors that play a key role in this binding. We did not detect the direct interaction between FAK and actins but the changes of cytoskeleton re-organization by characterizing microfilament bundling were indeed shown. We screened out the related mediator candidates that may play key roles in the regulation of cytoskeleton, and showed the result in a form of pheatmap and confirmed the changes of these candidates by qPCR. Through this process, our data finally presented such a regulation path from secreted extracellular protein (laminin β1)-focal adhension plaque (FAK)-cytoskeleton re-organization.

We also admitted that there were some limitations in our current study. Firstly, we detected the changes of secreted extracellular protein (laminin β1), focal adhension protein (FAK) and microfilaments (F-actin bundling) based on the changes of substrate stiffnesses. There were various types of forces including tensile, compressive, and shear stress, which may act on the physiological activities of chondrocytes at the same time^20,21^. We isolated chondrocytes and only showed the cell behavior changes in response to substrate stiffness. Thus, this cell behavior cannot fully reflect its physiological behaviors when considering its real living microenvironment. Secondly, we elucidated the mediation path based on the axis of laminin-FAK-microfilament. The biological mechanism of chondrocytes stimulated by microenvironment stiffness is a comprehensive regulation effect involving multi-signaling networks, and we only confirmed the importance of this single axis. Thus, the results shown in the current study could enlarge our understanding about the process of mechanosensing, mechanotransduction and cytoskeleton re-organization, but there are still many regulatory pathways that have not been revealed.

## Methods and materials

### Substrate preparation

In the current study, we used silicon-based elastomer polydimethylsiloxane (PDMS) to generate substrates with stiff and soft stiffnesses. PDMS substrate with high stiffness was fabricated by using 15 part of PDMS elastomer mixed with 1 part of curing agent Sylgard 184 (Corning, NY, USA) (Volume ratio: ~15:1), and PDMS substrate with low stiffness was fabricated by mixing 1 part of curing agent into 45 part of PDMS elastomer (Volume ratio: 45:1, PDMS elastomer vs. curing agent). We confirmed their physicochemical characterizations of these two PDMS substrates in the previous publications^8,14,32^. By curing the PDMS at the two ratios, we generated the substrates with high stiffness (15:1, ~450 kPa) and low stiffness (45:1, ~45 kPa) in the current study. Besides, when culturing cells, the surface of PDMS substrates needed to be coated with dopamine solution (0.12 mg·mL^−1^, w/v, in 1 mg·mL^−1^ Tris) in order to achieve hydrophilicity.

### Chondrocyte isolation

The tissue materials used in the current study were obtained according to the ethical principles, and the protocol about ethical principle was firstly approved before the experiments began by our Institutional Review Board (No.WCHSIRB-D-2017-029).

Chondrocytes were isolated from 0–3 days’ newborn mice (C57BL/6 J) as previously described^14^. The chondrocytes from hyaline cartilage of the knee joint were collected by 0.25% trypsin digestion for 30 min at 37 °C and 0.2% type II collagenase (sigma, MO) digestion for about 12 h at 37 °C till the cartilage tissue mass was completely digested. The isolated chondrocytes were filtered and cultured in 10% FBS DMEM (HyClone, Logan, UT). We used the chondrocytes at passage 1-2 (P1-P2).

### Cell seeding and cell sample preparation

When the stiff and soft substrates were prepared, chondrocytes were allowed to seed onto these substrates with 10% FBS DMEM for 12 h equilibration. Then we replaced 10% FBS DMEM with 2% fresh FBS DMEM for 12 h starvation. Next, we changed the culture media with 1% fresh FBS DMEM and the experiment started timing. It should be pointed out that the different experiments require different amounts of cells. At gene level, chondrocytes at the concentration of 1 × 10^6^ per well (35 mm single well, Corning) were needed for qPCR and RNA sequencing. At protein level, chondrocytes at the concentration of 0.5 × 10^6^ per well were needed for western blotting; chondrocytes at the concentration of 5000 per well were needed for immunofluorescence, and chondrocytes at the concentration of 5 × 10^6^ per well (60 mm single well) were needed for immunofluorescence.

### RNA sequencing

We used isolated chondrocytes at passage 2 for RNA sequencing. Briefly, chondrocytes (1 × 10^6^ per well) were seeded onto stiff and soft substrates for 72 h and harvested by trypsin digestion. the Trizol (No.15596-026, Thermo Fisher Scientific, Waltham, MA) was used for cell lysates. Then the cells were sent for RNA sequencing at Shanghai Lifegenes Biotechnology CO., Ltd (Shanghai, China) as previous described^8,31^. RNA concentration was detected (RNA Nano 6000 Assay Kit) prior to clustering of the index-coded samples by cBot Cluster Generation System (HiSeq 4000 PE Cluster Kit, Illumia, San Diego, CA). Raw data were obtained by matching reference genome using HISAT2 v2.1.0. Pheatmap was generated by online R package.

### siRNA interference

We used siRNA interference to show the influence of FAK on the cytoskeleton changes in chondrocytes. siRNA plasmid was from the Santa Cruz Biotechnology (sc-35353) and was transfected into chondrocytes by using Lipofectamine RNAiMAX (Invitrogen, Burlington, ON, Canada) according to the instruction from the manufacture. The final concentration of siRNA plasmid was ~100 nmol·L^−1^. The siCONTROL was set to be the scramble group.

### Scanning electron microscope (SEM)

Cells were seeded onto the stiff and soft substrates for 24 h and then fixed by 2.5% glutaraldehyde for 2–4 h. Then cells underwent gradient dehydration by using ethanol from 30%, 50%, 70%, 80%, 90% to 100%. Each gradient took 15 min. The cell samples were coated by a gold layer and then visualized by SEM.

### Quantitative real-time PCR (qPCR)

Before qPCR, the mRNA was purified and first strand cDNA was subsequently synthesized. The mRNA purification was performed by the Pure RNA Isolation Kit (RP5611, Bioteke Corporation, Peking, China). The first strand cDNA was synthesized by using RevertAid H Minus First Strand cDNA Synthesis Kit (No. K1632, Thermo Fisher Scientific). The primers used in the current study were listed in table S1. For qPCR, we used SYBR Green I-PCR master mix (SYBR® Premix Ex TaqTM II, No.RR82WR, TaKaRa, Tokyo, Japan) for PCR amplification (25 μl system). Each PCR sample contained 12.5 μl SYBR Green master mix, 1 μL cDNA, 1 μL forward primer, 1 μL reversed primer, 9.5 μL DDH_2_O. The reaction contained 45 cycles and each cycle included a process of 95 °C denaturation (5 min), 60 °C annealing (10–15 s) and 72 °C elongation(10–15 s). The fold changes were calculated using cycle threshold (∆∆CT) method. β-actin was used as the housekeeping gene (inner control).

### Western blotting

Cells seeded onto the PDMS substrates were treated with cell lysate buffer (RIPA lysis buffer, P0013B, Beyotime, Shanghai, China) as described^8,44^. Proteinase inhibitor solution PMSF (P7626, Shanghai, China) was additionally added at 1:100 ratio (v/v). After concentration quantification (BCA Protein Assay Kit, P0010, Beyotime, Shanghai, China), The samples were mixed with the sample buffer (Bio-Rad Laemmli sample buffer, Bio-Rad, Herciles, CA), boiled at 100 °C for 5 min and then stored at −20 °C till usage. We used 10% SDS-PAGE gels to separate target proteins and used PVDF membrane for immunoblotting. The primary antibodies used in the current study included: β-actin (1:1 000, sc-47778, Santa Cruz Biotechnology, Delaware Avenue), Laminin β1 (1:1 000, D9V6H, #13435, Cell signaling technology, Boston, MA), Laminin β2 (1:1 000, Cat: 384828, ZenBio, Chengdu, China), Laminin β3 (1:500, Cat: 822605, ZenBio, Chengdu, China), FAK (1:1 000, ab219363, Abcam, Cambridge, UK), PCNA (1:1 000, Cat: 200947-2E1, ZenBio). The secondary antibodies were included: Goat anti-mouse IgG (H&L) (HRP conjugate, 1:1 000, Cat: 511103, ZenBio), and Goat anti-rabbit IgG (H&L) (HRP conjugate, 1:2 000, Cat: 511203, ZenBio). The incubation times for primary antibodies were 12–16 h (overnight) at 4 °C and for secondary antibodies were 2 h at room temperature (RT). The blotting bands were captured by Immobilon ® Western (P90719, Millipore) with Bio-rad image system.

### Immunofluorescence and confocal laser scanning microscopy (CLSM)

The protocol was described as previously^8,32,45^. In brief, after being seeded on substrates for 72 h, cells were fixed by 4 % paraformaldehyde (PFA) for 10 min. Then, 0.25% Trition was used to permeabilize the cells for 5 min. After three times’ wash with 1 × PBS for 30 min, the cells were blocked with 0.5% bovine serum albumin (BSA) solution for 2 h. Then the cells were incubated with primary antibody (Laminin β1, 1:200 and FAK, 1:200) overnight (12–16 h) at 4 °C. After removal of primary antibody, the secondary antibody was incubated for 2 h at RT. Then we removed the antibodies and washed the cells with 1 × PBS three times, and added F-actin dye liquor (1:400, Phallotoxins, A12379, Alexa Fluor® 488 phalloidin, invitrogen, Carlsbad, CA) for overnight (12–16 h) at 4 °C. After removal of F-actin dye liquor, we used Dapi (10–100 μg·mL^−1^, D9542, Sigma-Aldrich, St. Louis, MO) for nuclear staining for 10 min. The immunofluorescent images were obtained by CLSM (Olympus, FV3000, Japan).

### Co-immunocoprecipitation (Co-IP)

Co-IP was performed by using Pierce co-immunoprecipitation Kit (lot no. SB240573B) as previously described^7,8^. Cells were allowed to grow to full confluence (>90%, cell number: >5 × 10^6^) and lyasted by IP Lysis/Wash buffer from the kit. The bait antibody FAK (10 μL × 0.98 μg·μL^−1^) was firstly bound to the resin and incubated with cell lysate for overnight (12–16 h). All preyed proteins were purified by centrifugal column provided by the kit according to the manufacturer’s instructions. We finally used elution buffer to collected ~50 uL total preyed proteins for western blotting detection. After concentration quantification by BCA, we performed western blotting to detect the potential binding proteins by using antibodies of laminin β1, 2, and 3. The immunoblotting was visualized by Immobilon ® Western (P90719, Millipore) with Bio-rad image system.

### Statistical analysis

We presented data as Means ± SD based on at least three independent experiments (*n* ≥ 3) in the current study. We consider the critical significance in each analysis was present when the threshold was less than 0.05 (*P* < 0.05). All the data were analyzed by two-tailed Student’s *t*-tests. The detailed statistical data were provided in the source data.

## Supplementary information

Supplementary figures and tables

Data source

## Data Availability

Any data generated in this study are available from the corresponding author upon request.
