# Supplementary material for: Microenvironmental stiffness mediates cytoskeleton re-organization in chondrocytes through laminin-FAK mechanotransduction
Source: Int J Oral Sci. 2022 Mar 11;14:15. doi: 10.1038/s41368-022-00165-5 (PMC8917190; doi:10.1038/s41368-022-00165-5)
Supplement: Supplementary file 1 — Supplementary figures and tables [file 41368_2022_165_MOESM1_ESM.doc]

Supplementary information for

**Microenvironmental stiffness mediates cytoskeleton re-organization in chondrocytes through laminin-FAK mechanotransduction**

**Supplementary figures**

**Figure S1**


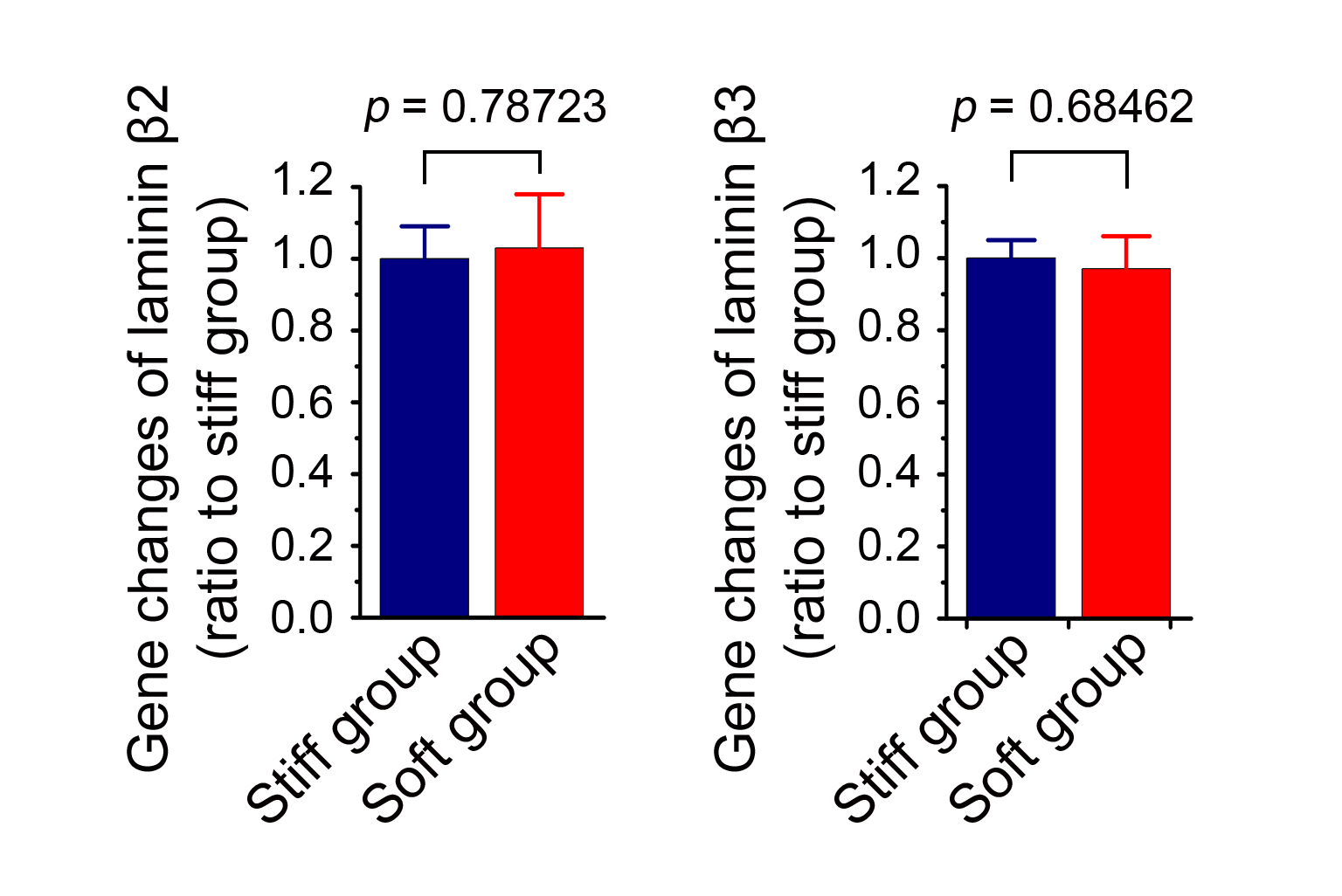


**Figure S1.** mRNA changes of laminin β2 and β3 by qPCR in chondrocytes in response to stiff and soft substrate stiffnesses. The results were based on three independent experiments (n = 3). The statistical analysis was based on two-tailed Student’s t-tests.

**Figure S2**


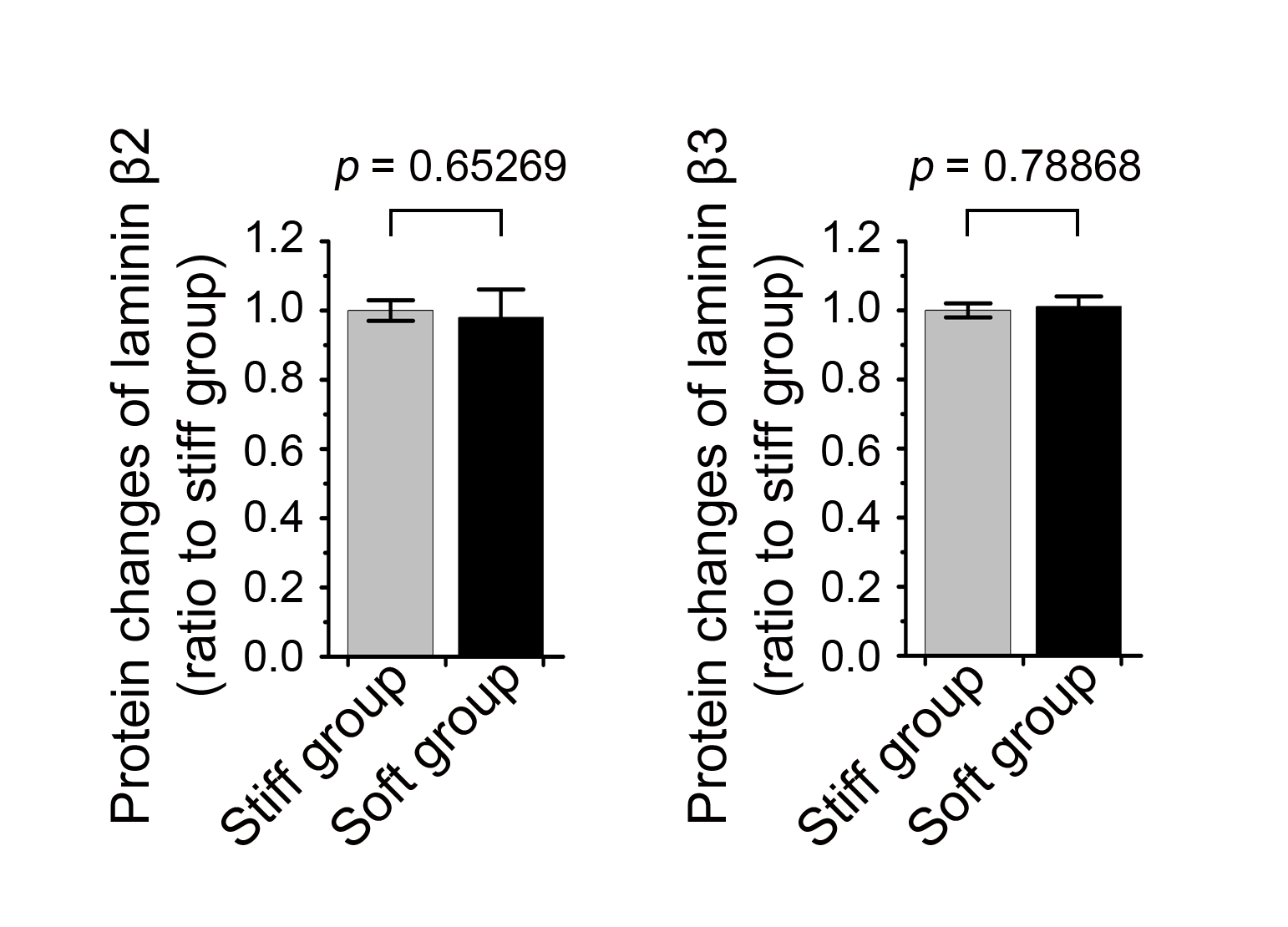


**Figure S2.** OD quantification of laminin β2 and β3 in chondrocytes in response to stiff and soft substrate stiffnesses. The results were based on three independent experiments (n = 3). The statistical analysis was based on two-tailed Student’s t-tests.

**Supplementary tables**

**Table S1. Primer pairs designed in the study (Mus musculus (house mouse))**

| Protein name | Gene name/gene ID | Primer pairs |
| --- | --- | --- |
| Glyceraldehyde-3-phosphate dehydrogenase (Candidate)  Actin, beta  Laminin B1  Laminin, beta 2  Laminin, beta 3  Focal adhesion kinase  or protein tyrosine kinase 2  Paxillin  Pleckstrin homology domain-containing, family A  Proline rich 5  Leiomodin 1  Growth associated protein 43  Myosin IA  Keratin 16  Serine/threonine kinase 33  PDZ and LIM domain 3  Endothelial cell surface expressed chemotaxis and apoptosis regulator  myosin light chain, phosphorylatable, fast skeletal muscle  CD93 antigen | GAPDH (110bp)  (NM_001289726.1)  β-actin (198bp)  (NM_007393.5)  Lamb1 (94bp)  (NM_008482.3)  Lamb2 (144bp)  (NM_008483.3)  Lamb3 (189bp)  (NM_001277928.1)  FAK/ PTK2 (82bp)  (NM_007982.2)  Pxn (192bp)  (NM_011223.3)  Plekha2 (105bp)  (NM_031257.3)  Prr5 (175bp)  (NM_146061.4)  Lmod1 (77bp)  (NM_053106.2)  Gap43 (100bp)  (NM_008083.2)  Myo1a (146bp)  (NM_001081219.2)  Krt16 (182bp)  (NM_001313958.1)  Stk33 (179bp)  (NM_054103.1)  Pdlim3 (154bp)  (NM_001374652.2)  Ecscr (85bp)  (NM_001368651.1)  Mylpf (118bp)  (NM_016754.5)  Cd93 (185bp)  (NM_010740.3) | Forward: GAGGGATGCTGCCCTTACC  Reverse: AAATCCGTTCACACCGACCT  Forward: TGAGCTGCGTTTTACACCCT  Reverse: GCCTTCACCGTTCCAGTTTT  Forward: TAGTTAAGCGACTTGACCCCCTTC  Reverse: AAATGTTGGGAGAGAGGAGAGG  Forward: CACGGTCGGGATGGAGTGG  Reverse: CAGCCAGGTACATCCAAGGAC  Forward: CTGATAAGCTGCTGGCGACT  Reverse: TCCCCCACAGGTGGATAACA  Forward: CGGACACATGCAGTCTCTGT  Reverse: TCGAGGGCATGGTGTATGTG  Forward: GAGCCTCTTGGATGAACTGGA  Reverse: CCCTGGGCCATGAACTTGAA  Forward: ACTTCTCCGAGGTTCAAGCA  Reverse: GGCTCTGTTCACATCACCCT  Forward: GCGAGCCATGGTTCACAG  Reverse: CTTCAACCTGCGGAGAGTCC  Forward: CTTTGCGCTCAAACCCACTC  Reverse: GGCTTGCTGGTGGTAGTAGG  Forward: GCTCAGCGGAGACAGAAAGT  Reverse: TCGGCTTGTTTAGGCTCCTC  Forward: GCCGGGACCCAGTTTAAGAA  Reverse: TATCGCGCCTGGACCATAAC  Forward: GGCGAGAATATCCACAGCTCC  Reverse: GTGAGGAGGCTCTAGGCAAAA  Forward: CAGCTTCTTTAGGTGTGGCGTT  Reverse: GTCACCCAGGAGCTTCATTTC  Forward: GACTGTGGCAGTGGACGTAG  Reverse: GCGTGTTAAACTGAGCGTGT  Forward: CATGAGGCTGGGTTCAGCAA  Reverse: TTCCTGCGAGGTCTGAGTTG  Forward: TACCACGGTATGTTAAGGGCTG  Reverse: CTTAGATCTCCTGGGGGCAA  Forward: GGGGAAACCGAGTAAGCCTC  Reverse: GCGAGGCTCATGGTTAGGAA |
